# Adoptive T-Cell Therapy in Advanced Colorectal Cancer: A Systematic Review

**DOI:** 10.1093/oncolo/oyab038

**Published:** 2022-02-19

**Authors:** Damie J Juat, Stephanie J Hachey, John Billimek, Michael P Del Rosario, Edward L Nelson, Christopher C W Hughes, Jason A Zell

**Affiliations:** Department of Molecular Biology & Biochemistry, University of California Irvine, Irvine, CA, USA; Department of Family Medicine, University of California Irvine, Irvine, CA, USA; Department of Family Medicine, University of California Irvine, Irvine, CA, USA; Division of Hematology/Oncology, Department of Medicine, University of California Irvine, Irvine, CA, USA; Chao Family Comprehensive Cancer Center, University of California Irvine, Irvine, CA, USA; Department of Molecular Biology & Biochemistry, University of California Irvine, Irvine, CA, USA; Division of Hematology/Oncology, Department of Medicine, University of California Irvine, Irvine, CA, USA; Chao Family Comprehensive Cancer Center, University of California Irvine, Irvine, CA, USA; Department of Molecular Biology & Biochemistry, University of California Irvine, Irvine, CA, USA; Department of Biomedical Engineering, University of California Irvine, Irvine, CA, USA; Division of Hematology/Oncology, Department of Medicine, University of California Irvine, Irvine, CA, USA; Chao Family Comprehensive Cancer Center, University of California Irvine, Irvine, CA, USA

**Keywords:** colorectal cancer, adoptive T-cell therapy, immunotherapy

## Abstract

Colorectal cancer (CRC) is the second leading cause of cancer-related deaths in the US. For the vast majority of patients with advanced CRC (ie, for those in whom metastatic tumors are unresectable), treatment is palliative and typically involves chemotherapy, biologic therapy, and/or immune checkpoint inhibition. In recent years, the use of adoptive T-cell therapy (ACT), leveraging the body’s own immune system to recognize and target cancer, has become increasingly popular. Unfortunately, while ACT has been successful in the treatment of hematological malignancies, it is less efficacious in advanced CRC due in part to a lack of productive immune infiltrate. This systematic review was conducted to summarize the current data for the efficacy and safety of ACT in advanced CRC. We report that ACT is well tolerated in patients with advanced CRC. Favorable survival estimates among patients with advanced CRC receiving ACT demonstrate promise for this novel treatment paradigm. However, additional stage I/II clinical trials are needed to establish the efficacy and safety of ACT in patients with CRC.

## Teaser

Colorectal cancer (CRC) is the second leading cause of cancer-related deaths in the US. In recent years, the use of adoptive T-cell therapy (ACT), leveraging the body’s own immune system to recognize and target cancer, has become increasingly popular. This review summarizes the current data for efficacy and safety of ACT in advanced CRC.

**Implications for Practice**

This systematic review summarizes the current data for the efficacy and safety of adoptive T-cell therapy (ACT) in advanced colorectal cancer (CRC). Available data suggest that ACT results in favorable overall survival (OS) and progression-free survival estimates when compared to currently available agents in the second/third-line setting for metastatic CRC and is generally well tolerated. However, additional stage I/II clinical trials are needed to establish the efficacy and safety of ACT in patients with CRC. Data emerging from novel clinical trials could improve survival outcomes among patients with advanced CRC by identifying new tolerable treatments that are able to control disease progression.

## Introduction

Colorectal cancer (CRC) is the second leading cause of cancer-related deaths in the US, and the third most commonly diagnosed type of cancer in both men and women.^1,2^ This high incidence rate is due in part to dietary and lifestyle factors, as well as general population aging.^3^

For localized (stage I) CRC, treatment involves surgery alone and surveillance monitoring with excellent outcomes. However, most patients with CRC present with locoregional (stages II-III) or advanced (stage IV) disease, partly because a limited proportion (50%-60%) of eligible individuals undergo recommended CRC screening. Standard treatment for regional stage colon cancer consists of surgical resection followed by chemotherapy such as FOLFOX (leucovorin, 5-fluorouracil, and oxaliplatin) or CAPOX (capecitabine, oxaliplatin). Rectal cancer is increasingly being treated with preoperative concurrent chemoradiation and/or chemotherapy (ie, total neoadjuvant therapy) prior to total mesorectal excision (TME)-based surgery.^4^ For the majority of patients with advanced CRC (stage IV), the first line of treatment involves a combination of chemotherapy plus a biologic agent (eg, vascular endothelial growth factor [VEGF]-inhibitor or epidermal growth factor receptor [EGFR]-inhibitor in Ras-wild-type patients).^5^ This standard-of-care chemotherapy and biologic regimen yields high disease control rates (DCRs) and improved disease-free survival; however, the treatments are not curative. Aside from the EGFR inhibitors among patients with Ras-wild-type tumors, and immune-checkpoint inhibition among a small proportion of patients with microsatellite instability-high (MSI-H) tumors, the majority of available treatments for advanced CRC are not patient tumor specific. Standard first-line advanced CRC treatments are associated with substantial toxicity (including fatigue, nausea/vomiting, anemia, leukopenia, thrombocytopenia, bleeding, hepatotoxicity, neurotoxicity, and hand-foot syndrome).^2,6^ Given that the 5-year survival rate for patients with stage IV CRC is only 12%,^7^ it is essential to identify new treatments that are tolerable, and able to control disease progression, which could improve overall survival (OS) among patients with CRC.

### Immunotherapy and Adoptive T-Cell Therapy

Cancer immunotherapy works by enhancing immune system recognition of the tumor. The use of immune checkpoint inhibitors, a type of immunotherapy, has resulted in practice-changing results for the field of Oncology and led to a Nobel Prize in Physiology or Medicine jointly to James P. Allison and Tasuku Honjo in 2018. Checkpoint inhibitor drugs work by either turning off an inhibitor mechanism that blocks cytotoxic T cells or blocking the tumor-associated immunosuppression that often develops in patients with substantial tumor burden, thereby allowing surrounding lymphocytes to attack the tumor without restraint. Consequently, the use of immune checkpoint inhibitors can also have autoimmune side effects.

In advanced CRC, clinical benefits of immune checkpoint therapy are currently limited to the small proportion of advanced patients (approximately 5%) demonstrating a high level of microsatellite instability (MSI-H).^8,9^ MSI-H CRC is associated with both high rates of tumor mutation, and tumor-infiltrating lymphocytes (TILs), which explains immune checkpoint inhibitor therapy in MSI-H CRC. Currently, these promising immune checkpoint inhibitor agents are ineffective and therefore not used in 95% of patients with metastatic CRC outside of the clinical trial setting, highlighting an unmet clinical need. Myriad factors contribute to clinical failure of immunotherapies, which, while interdependent, can be broadly categorized by (1) a lack of immune cell penetration into the tumor, (2) immunosuppression of immune cells that do penetrate the tumor, and (3) an inability of immune cells to target the heterogeneous cellular populations within a tumor.^10-13^ Some approaches taken to increase the efficacy of immune checkpoint inhibitor efficacy in CRC are promising, such as combination chemotherapy and biologic therapy together with monoclonal programmed cell death ligand-1 (PDL1) antibodies as done using atezolizumab in the in the randomized phase II AtezoTRIBE trial.^14^

While several approaches to overcoming these obstacles are being studied in immunotherapy, this review focuses on the potential therapeutic effectiveness of adoptive T-cell therapy (ACT). In contrast to immune checkpoint approaches, ACT uses patient-derived T cells expanded ex vivo that are then reinfused into patients.^15^ ACT has the potential to improve or replace current treatments and has been shown to have an 88% complete response (CR) rate in a subset of patients living with cancer with hematological diseases.^16^ However, few patients with CRC benefit from immunotherapy, potentially due to limited access of the T cells to solid tumors, immune cell evasion mechanisms, and/or tumor heterogeneity.^17^

In general, adoptive cell therapy or cellular immunotherapy uses cells from the immune system, either the patient or a donor, to eliminate cancer. T cells are part of the body’s adaptive immune system and fall mostly into 2 broad subsets, CD4+ and CD8+ T cells. CD4+ T cells (helper T lymphocytes) function in a supporting role to the adaptive immune response, primarily through cytokine production that tailors the immune response to different classes of pathogens. CD8+ cytotoxic T lymphocytes (CTLs) are activated in response to tumor-associated antigens (TAA) presented in the context of major histocompatibility complex (MHC) class I molecules. T cells express either an αβ or γδ T-cell receptor heterodimer. Although most T cells are from the αβ T-cell lineage (95%), notably, γδ T cells are one of the most prominent immune cells in the gut^18,19^ and may therefore be good candidates for immunotherapeutic strategies in CRC.

T cells used in ACT can be derived in several ways. For example, TILs are collected from surgically resected tumors and, as such, are naturally targeted to the tumor. After collection, TILs can be re-activated, expanded, and re-infused into the patient. Alternatively, T cells can be collected from peripheral blood and genetically modified to display activity against tumor cells. In T-cell receptor therapy (TCR), T cells are equipped with a new T-cell receptor that targets a specific TAA presented by an MHC molecule.^20^ For patients living with CRC, carcinoembryonic antigen is a common target antigen because it is frequently upregulated in this cancer.^21^ MHC-mediated killing such as this requires autologous T cells (from the patient) or MHC-matched T cells from donors. Finally, cytokine-induced killer (CIK) cells are a type of cytotoxic T cell that can kill in the absence of TCR-MHC interaction and thus, overcome problems associated with MHC restriction.^22^ Although they have both the characteristics of T cells and NK cells, CIKs are treated as a type of T cell in this review.

Another type of genetically modified T cell is the chimeric antigen receptor T cell or CAR-T cell. CAR-T cells are valuable because, like CIK cells, they can bypass the MHC system and directly target an antigen of interest.^23^ However, this recognition is limited to surface-expressed antigens. CAR-T cell therapy has shown great success in hematologic diseases such as leukemia or lymphoma; it has unfortunately elicited a response rate of only 9% in solid tumors, and even less in patients with CRC.^24^ This poor therapeutic response is due in part, to the lack of a target antigen that is both uniformly and strongly expressed on CRC as well as possible poor penetration of solid tumors. However, the field is rapidly changing as data emerge from novel clinical trials. In this study, we set out to perform a systematic review of the literature to provide a current assessment of the safety and efficacy of ACT in advanced CRC.

## Methods

### Search Strategy and Selection Criteria

The objective of this literature review is to assess the efficacy and safety of ACT in patients living with advanced CRC. The search was limited to studies published within the past 10 years with an English language restriction. The start date of 2010 was chosen to coincide with the publication of the seventh American Joint Committee on Cancer (AJCC) Staging Manual.^25^

The Preferred Reporting Items for Systematic Review and Meta-Analysis guidelines were used to conduct and report this systematic review.^26^ A search was conducted of Cochrane Library, Web of Science, SCOPUS, and Medline PubMed using the following search terms: colon cancer, colorectal neoplasm, colonic neoplasm, immunotherapy, and adoptive cellular immunotherapy. Following the search, all identified citations were collated and uploaded into EndNote X9 2019, and duplicates removed. The reference lists of the included studies were also manually searched for other eligible studies.

### Eligibility

Studies meeting the following inclusion criteria were included in the review: (1) randomized controlled trials, quasi-experimental studies, cohort studies, case series, and case reports; (2) the study participants were 18 years of age or older with histologically confirmed, stages III and IV metastatic CRC defined by the seventh AJCC Staging Manual; and (3) the study participants had previously received first-line treatment for advanced CRC including surgery, chemotherapy, and radiotherapy. There were no limitations related to the length of the intervention and study duration, which maximized the studies eligible for review.

### Interventions

- ACT in combination with chemotherapy versus supportive care, no treatment, or placebo
- ACT in combination with chemotherapy versus chemotherapy alone
- ACT in combination with chemotherapy

### Outcome Measures

#### Primary

- OS: the interval between the date of starting ACT and the date of death from any cause, OS time frame determined by each study follow-up.
- Progression-free survival (PFS): according to the universally accepted World Health Organization (WHO) or *Response Evaluation Criteria in Solid Tumors* (RECIST) guidelines.
- Objective response rate (ORR): according to RECIST guidelines, the proportion of patients who have a partial or CR to therapy.
- DCR: according to RECIST guidelines, the proportion of patients who have a partial or CR to therapy including those with stable disease (SD).

#### Secondary

- Survival rates: proportion of participants in a study who were still alive at 3 months, 6 months, 1 year, and 2 years.
- PFS rates: proportion of participants in a study who did not have disease progression at 3 months, 6 months, 1 year, and 2 years.

Treatment-related adverse events: defined by WHO, Eastern Clinical Oncology Group, National Cancer Information Center-Common Toxicity Criteria, and Common Terminology Criteria for Adverse Events.

## Results

### Study Selection

**Figure 1.**
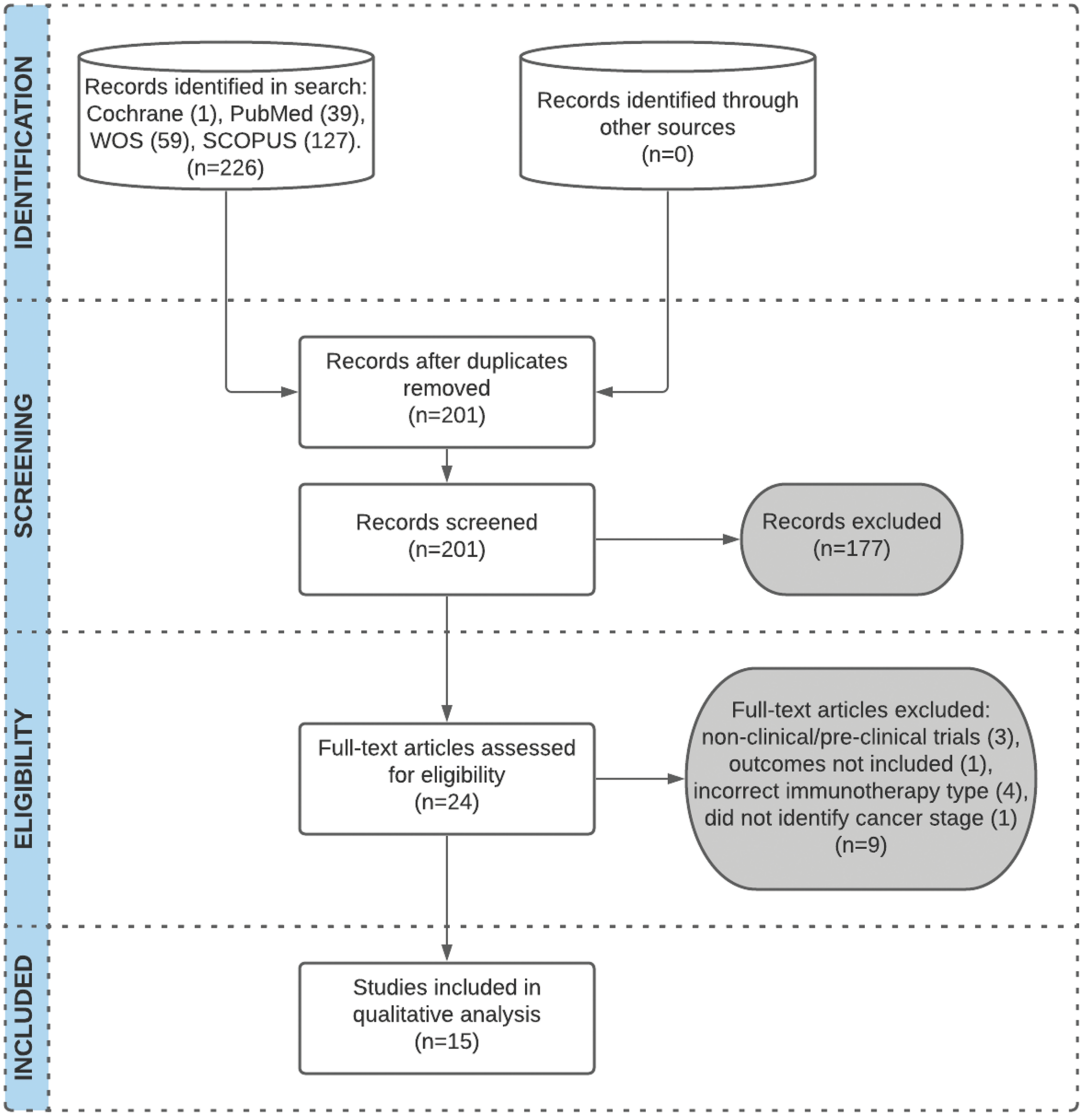
PRISMA flow diagram. Fifteen studies were included in this review.

The electronic database searches identified 15 studies for inclusion (Fig. 1). Initial search of the Cochrane Library, PubMed, Scopus, and Web of Science yielded 226 studies (Table 1). After the removal of duplicates (25) and screening for unrelated articles/articles published prior to 2010 (177), 24 full-text articles were assessed. The final 15 articles were selected for inclusion in this review following the exclusion of 9 articles (eg, incorrect immunotherapy type, nonclinical/preclinical trials, outcomes not included in results, did not separate outcome data by cancer stage).

**Table 1.** Search terms for systematic review.^a^

Database	Search terms
PubMed	((colon cancer OR Colorectal Neoplasms OR Colonic Neoplasms OR colorectal cancer)AND (car-t cell therapy OR immunotherapy, adoptive OR adoptive cellular immunotherapy) AND (clinicaltrial[Filter] OR multicenterstudy[Filter] OR randomizedcontrolledtrial[Filter])) OR (((colon cancer OR Colorectal Neoplasms OR Colonic Neoplasms OR colorectal cancer) AND (car-t cell therapy OR immunotherapy, adoptive OR adoptive cellular immunotherapy)) AND (cohort OR random∗ or “clinical trial”))
Cochrane Library	(“colon cancer” OR “Colorectal Neoplasm∗” OR “Colonic Neoplasm∗”) AND (“car-t cell therap∗” OR “immunotherapy, adoptive” OR “adoptive cellular immunotherap∗”)
Web of Science	ALL FIELDS: ((colon cancer OR Colorectal Neoplasms OR Colonic Neoplasms OR colorectal cancer) AND (car-t cell therapy OR immunotherapy, adoptive OR adoptive cellular immunotherapy)) Refined by: LANGUAGES: (ENGLISH) AND TOPIC: (clinical trial)
SCOPUS	TITLE-ABS-KEY ((“*colon cancer” OR* *“Colorectal Neoplasm∗”* OR *“Colonic Neoplasm∗”*) AND (*“car-t cell therap∗”* OR *“immunotherapy, adoptive”* OR *“adoptive cellular immunotherap∗”*)) AND (LIMIT-TO ( LANGUAGE,*“English”*))

a Searched databases were PubMed, Cochran Library, Web of Science, and SCOPUS. The initial literature search was performed on May 8, 2020.

A total of 15 ACT clinical trials were analyzed and 8 of the 15 trials were formally registered on ClinicalTrials.gov and UMIN Clinical Trial Registry (Table 2). The types of ACTs included were TIL (*n* = 3), CAR-T (*n* = 3), CIK (*n* = 4), αβ T cell (*n* = 2), γδ T cell (*n* = 2), and TCR (*n* = 1) therapy. The average number of participants was 7, with a range from 1 to 21. All studies included were non-randomized studies and consisted of phase I (*n* = 8), phase Ib (*n* = 1), phase Ib/II (1), phase I/II (*n* = 2), and retrospective trials (*n* = 3). Most of the included studies were based in China (*n* = 7) and the remaining in Japan (*n* = 3), US (*n* = 3), Sweden (*n* = 1), and Australia (*n* = 1).

**Table 2.** Overview of included ACT trials.

No.	Phase	Publication year ^a^	Country	Institution	Trial registration	Cell type	Number of participants	Received cell product	Additional treatment given^b^
1	I	2013^27^	Japan	University of Tokyo Hospital	UMIN000000854	γδ T-cell	6	6^c^	None
2	Ib/II	2018^28^	China	Chinese PLA General Hospital	NCT01799083	CIK	4	4^d^	Decitabine + previous first-line line chemotherapy
3	Ib	2010^29^	Sweden	Karolinska University Hospital	—	TIL	11	11	5-Fluorouracil/leucovorin
4	I	2015^30^	USA	Roger Williams Medical Center	NCT01373047	TIL	6^e^	5^e^	Interleukin-2
5	I/II	2010^31^	USA	National Institutes of Health	NCI-09-C-0041	Anti -ERBB-2 CAR-T	1	1	Interleukin-2
6	I	2011^32^	Australia	University of Queensland	—	γδ T cells	3	3	None
7	I	2011^33^	US	National Institutes of Health	NCT00923806	Anti-CEA TCR	3	3	Interleukin-2
8	I	2019^34^	China	Capital Medical University Cancer Center	NCT03757858	CIK	7	7	Pembrolizumab or chemotherapy^f^
9	I	2018^35^	China	Chinese PLA General Hospital	NCT02541370	Anti-CD133 CAR-T	2	2	Nab-paclitaxel
10	-	2016^36^	Japan	Fukuoka University Faculty of Medicine	—	αβ T-cell	15	15	CAPOX plus bevacizumab
11	I	2017^37^	Japan	Fukuoka University Faculty of Medicine	UMIN000010908	αβ T-cell	6	5^g^	CAPOX plus bevacizumab
12	I	2017^38^	China	Third Military Medical University	NCT02349724	Anti-CEA CAR-T	10	10	CTX
13	-	2015^39^	China	Guangdong Provincial Hospital of Chinese Medicine	—	CIK	5	5	None
14	I/II	2015^40^	China	The Affiliated Hospital of Guiyang Medical College	—	TIL	25^h^	9^h^	5-Fluorouracil-based chemotherapy
15	—	2013^41^	China	Guangdong Provincial Hospital of Chinese Medicine	—	CIK	21	21^i^	None

a Citation number.

b Treatment regimens were given to select patients in each trial as determined by trial investigators.

c All 6 patients received the cell product; however, the number of cells infused varied between them.

d All 4 patients enrolled received at least 2 doses of the cell product.

e Six patients with colon cancer were enrolled in the study but one withdrew before completing the treatment protocol due to extrahepatic disease progression prior to his third CAR-T dose.

f Salvage chemotherapy included paclitaxel/carboplatin, oxaliplatin/capecitabine, or nanoparticle albumin-bound paclitaxel.

g 5/6 patients received at least 6 cycles (up to 23 cycles) of infusion of the cell product, while one patient discontinued treatment after 4 cycles. This patient was included in subsequent survival analyses.

h Twenty-five patients with stage IV CRC were enrolled, but cell product was only successfully generated from 9 patients. The 16 patients for which cell product could not be generated were considered as “control” group.

i Twenty-one patients received at least one dose of cell product, although the number of infusions and timing of infusion were variable. While stated that one patient from the treatment group withdrew, it appears that all 21 patients who were intended to be treated were included in survival analyses.

ACT, adoptive T-cell therapy; CAPOX, capecitabine plus oxaliplatin; CAR-T cell, chimeric antigen receptor T cell; CIK, cytokine-induced killer; CTX, cyclophosphamide; TIL, tumor-infiltrating lymphocytes.

### Patient Characteristics

A total of 108 patients were enrolled across all trials (Table 3). The median age (data available for 80 out of 108 patients) was 62 years. The age range of the patients was 33 to 82 years. Most patients included had stage IV CRC (73%) and the remaining patients were diagnosed with stage III (15%), stage I/II (5%), or not specified within the article (7%). Regarding tumor site, the colon was the most common site, accounting for 68% of patients followed by the rectum for 24% of patients. Several metastatic locations were prevalent among enrolled patients with the liver being most common (33%). ACT was used as a second-line treatment or above in 72% of patients and the patients that did not yet receive first-line treatment (28%) were given chemotherapy during the trial. Chemotherapy regimens varied across all patients; however, most were prescribed FOLFOX/CAPOX (Table 4).

**Table 3.** Patient characteristics.

Characteristics	*n* (%)
Total patients	108
Gender	
Male	47 (44)
Female	54 (50)
Not specified	7 (5)
Age, median (range)^a^	62 (33-82)
* *Not specified	28 (26)
AJCC stage	
I/II^b^	5 (5)
III	16 (15)
IV	79 (73)
Not specified	8 (7)
Tumor site	
Colon	73 (68)
Rectum	26 (24)
Not specified	9 (7)
Location of metastases^c^	
Liver	36 (33)
Lungs	26 (24)
Bone	4 (4)
Lymph nodes	19 (18)
Spleen	1 (1)
Not specified	46 (43)
Previous lines of treatment	
None	30 (28)
One or more	69 (64)
2 or more	9 (8)

a Data available for 80 patients.

b Some articles did not separate data by AJCC stage, some stage I/II data included.

c Some patients had multiple sites of metastasis.

AJCC, American Joint Committee on Cancer.

**Table 4.** Chemotherapy regimens.

Chemotherapy	Previous, *n* (%)	Concurrent, *n* (%)
None	21 (19)	32 (30)
FOLFOX/CAPOX	35 (32)	23 (21)
FOLFIRI	11 (10)	3 (3)
5-FU	3 (3)	15 (14)
Tegafur-Uracil/S-1	4 (4)	—
Irinotecan	—	2 (2)
Cyclophosphamide	—	13 (12)
Fludarabine	—	11 (10)
Cisplatin	—	1 (1)
Etoposide	—	1 (1)
Paclitaxel	—	2 (2)
Capecitabine	1 (1)	—
N-P^a^	1 (1)	—
Not specified	68 (63)	14 (13)

Note: Some patients had multiple chemotherapy regimens.

a Vinorelbine and cisplatin.

### Treatment Response

Out of 11 articles that presented treatment response data, most patients were recorded to have SD according to RECIST guidelines with DCRs ranging from 17% to 100% (Table 5). Two of these trials recorded high ORRs, 80% and 83% using αβ T-cell therapy. Both trials used a dose of 5 × 10^9^ αβ T lymphocytes cultured ex vivo on day 17 or 18 and once every 3 weeks afterward for 4.5 months. These studies were also published by the same author.^36,37^ Two additional studies reported patients with CR to ACT using TIL and CIK therapy.^29,34^ Lastly, 5 out of 11 studies recorded patients with disease progression.^30,32-34,38^ Two articles recorded low DCR rates of 0% and 33% using γδ T cells and TCR, respectively.^32,33,42^ In the article with a DCR rate of 0%, the average dose per infusion was 1.7 × 10^9^ γδ T cells.^32^ This is an approximately 10-fold higher dose than is typically infused for CAR T-cell products (3 × 10^6^ CAR T cells/kg or 1.9 × 10^8^ cells for the average person), suggesting that the lack of efficacy was not due to low cell numbers.

**Table 5.** Treatment response (RECIST).

No.	*n*				ORR, %	DCR, %	Censored, *n*	Total patients, *n*
	CR	PR	SD	PD				
1	—	—	—	—	—	—	—	—
2	0	0	4	0	0	100	0	4
3	4	1	5	0	46	91	1	11
4	0	0	1	4	0	17	1	6
5	—	—	—	—	—	—	—	—
6	0	0	0	3	0	0	0	3
7	0	1	0	2	33	33	0	3
8	2	1	2	1	43	71	0	7
9	0	0	2	0	0	100	0	2
10	4	8	3	0	80	100	0	15
11	2	3	1	0	83	100	0	6
12	0	0	7	3	0	70	1	10
13	0	0	5	0	0	100	0	5
14	—	—	—	—	—	—	—	—
15	—	—	—	—	—	—	—	—

CR, complete response; DCR, disease control rate; ORR, objective response rate; PR, partial response; RECIST, Response Evaluation Criteria in Solid Tumors; SD, stable disease.

### Progression-Free Survival and OS

Out of 7 articles that presented PFS data, the median PFS ranged between 5.5 and 17.5 months (Table 6). The 1-year PFS rate ranged between 25% and 89.5%. OS was recorded in 5 articles and the median ranged between 4.5 and 16.5 months (Supplementary Appendix 1). The 1-year OS rate ranged between 20% and 100%. One patient from a case report had an overall survival of 0.16 months due to a fatal (grade 5) adverse event in response to CAR-T infusion.

**Table 6.** Progression-free survival.

No.			PFS rate, *n* (%)				Total patients, *n*
	PFS median (range)	Censored, *n*	3 months	6 months	1 year	2 years	
1	6.1 (0.6-35)	1	5 (83)	4 (67)	2 (33)	1 (20)	—
2	6.5 (3-29)	0	3 (75)	2 (50)	1 (25)	1 (25)	4
3	12 (6-36)	6	4 (80)	4 (80)	2 (40)	1 (20)	11
4	—	—	—	—	—	—	6
5	—	—	—	—	—	—	—
6	—	—	—	—	—	—	3
7	5.5 (5-6)	1	2 (100)	—	—	—	3
8	—	—	—	—	—	—	7
9	—	—	—	—	—	—	2
10	17.5 (7.5-28)	5	15 (100)	15 (100)	11 (79)	3 (25)	15
11	15 (8.3-21.6)	2	6 (100)	6 (100)	4 (67)	2 (33)	6
12	—	—	—	—	—	—	10
13	12 (5-24)	0	5 (100)	4 (80)	2(40)	—	5
14	—	—	—	—	—	—	—
15	—	1	—	—	− (89.5)	− (59.65)	21

Note: Includes DFS and RFS.

DFS, disease-free survival; PFS, progression-free survival; RFS, recurrence-free survival.

### Serious Adverse Events

Adverse events were well reported across most of the selected studies and are summarized in Supplementary Appendix 2. One patient who was infused with 1 × 10^10^ anti-ERBB2 CAR-T suffered several grade 4 adverse events including gastrointestinal bleeding and pulmonary edema. This patient ultimately died from cardiac arrest. Adverse event data are not shown from 4 studies because they did not differentiate event occurrence between cancer stage or cancer type.

## Discussion

This systematic review presents the current clinical landscape for ACT in advanced CRC. Currently, no phase III clinical trials of ACT have been resulted in advanced CRC. Available data in early phase (ie, phase I, phase II) clinical trials suggest that ACT may result in prolonged OS and PFS, and is generally well-tolerated among patients with advanced CRC.

The natural course of disease for metastatic CRC in patients who have stopped responding to standard treatment is generally poor. To illustrate this, one can examine results of modern clinical trials for metastatic CRC, where a placebo group was included (ie, a group representing the “natural progression of disease”). Two previously published clinical trials, CORRECT and RECOURSE, tested monotherapies including regorafenib and TAS-102, respectively, in patients with chemo-refractory CRC (ie, patients treated in the “third-line setting”). Each reported similar median OS rates for the placebo arms (no therapy) at 5 and 5.3 months, respectively.^43,44^ In CORRECT, regorafenib as mCRC treatment in the third-line setting resulted in a statistically significant difference in median OS (6.4 months vs 5 months for placebo) and significant but small difference in PFS (1.9 vs 1.7 months). In the RECOURSE trial of third-line therapy for mCRC, TAS-102 monotherapy versus placebo resulted in statistically significant improvements in OS (7.1 months vs 5.3 months) and PFS (2.0 months vs 1.7 months). Another important study done in the second-line setting, RAISE, which included chemotherapy as their control group (5-fluorouracil, leucovorin, irinotecan, “FOLFIRI”) without ramucirumab reported a median OS of 11.7 months and median PFS of 4.5 months.^45^ The higher median OS and PFS rate in RAISE compared with CORRECT and RECOURSE is due to RAISE not having a true placebo arm (and reveals the effects of a standard chemotherapy regimen in the second-line treatment setting, that is, patients who have received just one prior line of treatment). Of note, the interventional arm in RAISE (FOLFIRI + ramucirumab) attained a median OS estimate of 13.3 months. These findings, among others, highlight the need to develop effective therapeutic strategies for patients with advanced CRC. Here, these results serve as a historical comparison from which to place the selected mCRC ACT clinical trial results into context.

In this review, 4 out of 5 articles reporting OS had extended median estimates exceeding 5 months and 3 out of 5 reported median OS exceeding 14 months. All 7 articles that reported PFS had median estimates exceeding 4.5 months. It is encouraging that the PFS and OS estimates from these studies, involving heavily pretreated patients with metastatic CRC, surpassed the median OS and PFS of the placebo control arms of CORRECT and RECOURSE, suggesting that the treatments are active. Specifically, since patients will likely use ACT as a second or third line of treatment, it is encouraging that the studies in this review reveal PFS and OS estimates that are comparable, if not superior to both the active and control arms in RAISE, a modern second-line clinical trial for metastatic CRC (ie, representing a current standard of care).

To place the toxicity results from this systematic review into perspective, we once again turn to the clinical trials literature. Clinical trial “CALGB-80405” examined FOLFOX6 and FOLFIRI with either bevacizumab or cetuximab in patients with Ras-wild type mCRC as first-line treatment, revealing an severe adverse event (SAE) incidence rate of 53% (ie, events grade 3 or higher).^46^ In comparison, only 30% of patients receiving ACT on protocol experienced an adverse event of grade 3 or higher according to this review, in a population of patients who have received multiple prior treatments (ie, a population at greater risk for treatment-related adverse events). This suggests that ACT tolerance compares favorably to current chemotherapy-based standards of care.

One patient in this review suffered treatment-related death during a clinical trial studying anti-ERBB2 CAR-T therapy. She was diagnosed with Her2+ colon cancer that metastasized to her liver and 6 lymph nodes. After several chemotherapy regimens, 1 × 10^10^ CAR-T cells targeting ERBB2, which has been found to be overexpressed in several different cancers including colon cancer, were used as the fourth line of treatment. Within a few hours after the first infusion, the patient had respiratory distress due to pulmonary edema and after 12 hours, the patient developed severe hypotension and experienced 2 cardiac arrests. After 5 days, the patient suffered from progressive hypotension, bradycardia, and gastrointestinal bleeding that eventually led to cardiac arrest and treatment-related death.^31^ This clinical trial has since been terminated (the above patient was the first and only patient receiving the investigational ERBB2 CAR-T therapy). While most patients did not experience severe adverse events, it is important to note that ACT is still being optimized and developed. After postmortem analysis, the researchers in this study concluded that the patient’s death was a result of transferring highly active anti-ERBB2 T cells that recognized ERBB2 expressed by normal lung tissue. This triggered a release of inflammatory cytokines (TNF-α and IFN-γ) causing pulmonary toxicity and edema followed by a rapid surge of cytokines (also called cytokine release syndrome or CRS) ultimately resulting in multiorgan failure. Post-mortem analysis also revealed hemorrhagic microangiopathic injury and generalized rhabdomyolysis. While not specifically mentioned by the authors, another consideration is HER2 (ERBB2) expression in the myocardium and myoendothelium, a known factor in anti-Her2 trastuzumab cardiac toxicity.^47,48^ CRS is a side effect of immunotherapy that causes widespread activation of the immune system. One way to minimize this effect is the use of cytokine-blocking drugs such as tocilizumab and siltuximab (anti-IL-6R and anti-IL-6, respectively). Dexamethasone, an anti-inflammatory drug, was used to help combat the adverse reaction to CAR-T infusion; however, the cytokine-blocking drugs that were available at the time of this clinical study were not used.^49^ Additionally, severe adverse reactions such as CRS could be avoided by restricting the dose of active T cells. Lastly, other CAR-T studies included in this review targeted other specific antigens including CEA and CD133. More research is needed to determine whether targeting these antigens would be successful in patients with CRC. This again highlights the need to direct research efforts toward an accurate and high-throughput method for testing ACT.

Among metastatic CRC among patients with MSI-H tumors (ie, representing just 5%-7% of patients with metastatic CRC), high responses have been documented with immune checkpoint inhibition, which is generally well tolerated.^8^ More recently, single agent anti-programmed cell death protein-1 (PD-1) therapy with pembrolizumab^50^ or combination immune checkpoint therapy against CTLA4 (ipilimumab) and PD-1 (nivolumab) has been established as effective first-line regimens for patients with MSI-high metastatic CRC.^51^ In our systematic review, the proportion of patients with MSI-H/MSS tumors was not reported; however, it would be an important consideration for the stratification of patients in future studies.

Additional stage I/II clinical trials are needed to truly understand the efficacy and safety of ACT in patients with CRC. Currently, there are 2 phase I clinical trials for ACT in patients with CRC that are either recruiting or will be recruiting patients. One trial (NCT04107142) is a dose escalation trial for CAR-T cells targeting NKG2DL thought to be important in the regulation of tumor progression, and the other (NCT03970382) is testing neoantigen targeted TCR on locally advanced or metastatic tumors.^52^ One other trial is active (NCT02757391) and is testing a CD8+ T-cell therapy with pembrolizumab, the immune checkpoint PD-1 inhibitor, while the remaining 2 other trials registered on ClinicalTrials.gov were terminated due to feasibility and sponsor decision.

### Study Limitations

The review had several limitations. First, all studies included in this review were non-randomized trials (ie, there are no completed phase III trials published to date), which makes it difficult to compare the results between experimental groups. Second, some studies included IL-2, hyperthermia, and anti PD-1 which may confound the overall results. Lastly, due to the low sample size of each study, it was not feasible to combine results for statistical analysis.

## Conclusion

This review examined the current clinical landscape of ACT in patients with CRC. Median survival estimates for mCRC treated with standard approved therapies in the second/third-line setting are just 5 months OS and 1.7 months PFS.^43-45^ Three of 5 ACT trials reported median OS estimates exceeding 14 months. All 7 ACT trials reported PFS median estimates exceeding 4.5 months. Favorable ACT trial OS and PFS estimates suggest promise for this new treatment paradigm. ACT appears to be well tolerated in patients with advanced colon cancer.

## Supplementary Material

oyab038_suppl_Supplementary_Material

## Data Availability

The data underlying this article will be shared on reasonable request to the corresponding author.
