# Supplementary material for: Adoptive T-Cell Therapy in Advanced Colorectal Cancer: A Systematic Review
Source: Oncologist. 2022 Feb 19;27(3):210–9. doi: 10.1093/oncolo/oyab038 (PMC8914488; doi:10.1093/oncolo/oyab038)
Supplement: oyab038_suppl_Supplementary_Material [file oyab038_suppl_supplementary_material.pdf]

The Oncologist
The official journal of the Society for Translational Oncology

Supplemental Appendix for:
Adoptive T-Cell Therapy in Advanced Colorectal Cancer: A Systematic Review
Jason Zell et al.

Appendix 1. Overall Survival

			OS Rate, n (%)				
**#**	**Overall Survival, median (range)**	**Censored, n**	**3 months**	**6 months**	**1 year**	**2 years**	**Total, n**
**1**	-	-	-	-	-	-	-
**2**	14.5 (3-19)	0	3 (75)	3 (75)	2 (50)	-	4
**3**	14 (6-40)	6	5 (100)	3 (60)	2 (40)	1 (20)	11
**4**	4.5 (3.3- 23)	1	5 (100)	2 (40)	1 (20)	-	6
**5**	-	-	-	-	-	-	-
**6**	-	-	-	-	-	-	3
**7**	-	-	-	-	-	-	3
**8**	-	-	-	-	-	-	7
**9**	-	-	-	-	-	-	2
**10**	-	-	-	-	-	-	15
**11**	16.6 (13.3-31.7)	3	6 (100)	6 (100)	6 (100)	1 (17)	6
**12**	-	-	-	-	-	-	10
**13**	-	-	-	-	-	-	5
**14**	8.5 (5-28)	5	9 (100)	7 (88)	5 (63)	4 (57)	-
**15**	-	-	-	-	-	-	

Appendix 2. Adverse Events	n (%)		
	Grade 3	Grade 4	Grade 5
**Total**	14 (28)	1 (2)	1 (2)
**Abdominal pain**	2 (4)	0 (0)	0 (0)
**Alkaline Phosphatase**	2 (4)	0 (0)	0 (0)
**Anemia**	1 (2)	0 (0)	0 (0)
**Anorexia**	1 (2)	0 (0)	0 (0)
**AST**	1 (2)	0 (0)	0 (0)
**Bilirubin**	1 (2)	0 (0)	0 (0)
**Cardiac Arrest**	0 (0)	1 (2)	1 (2)
**Colitis**	1 (2)	0 (0)	0 (0)
**Diarrhea**	3 (6)	0 (0)	0 (0)
**Emesis**	3 (6)	0 (0)	0 (0)
**Fatigue**	1 (2)	0 (0)	0 (0)
**Fever**	3 (6)	0 (0)	0 (0)
**Gastrointestinal bleeding**	0 (0)	1 (2)	0 (0)
**Hypertension**	2 (4)	0 (0)	0 (0)
**Hypotension**	0 (0)	1 (2)	0 (0)
**Leukopenia**	1 (2)	0 (0)	0 (0)
**Neutropenia**	1 (2)	0 (0)	0 (0)
**Pulmonary Edema**	0 (0)	1 (2)	0 (0)
**Subscapular Hematoma**	1 (2)	0 (0)	0 (0)
**Tachycardia**	1 (2)	0 (0)	0 (0)
